# Proof of Concept of a Binary Blood Assay for Predicting Radiosensitivity

**DOI:** 10.3390/cancers13102477

**Published:** 2021-05-19

**Authors:** Sophie Deneuve, Céline Mirjolet, Thierry Bastogne, Mirlande Duclos, Paul Retif, Philippe Zrounba, Pierre-Eric Roux, Marc Poupart, Guillaume Vogin, Nicolas Foray, Sandrine Pereira

**Affiliations:** 1 Centre Léon Bérard, UNICANCER, 69008 Lyon, France; sophie.deneuve@lyon.unicancer.fr (S.D.); philippe.zrounba@lyon.unicancer.fr (P.Z.); pierre-eric.roux@lyon.unicancer.fr (P.-E.R.); marc.poupart@lyon.unicancer.fr (M.P.); 2 Radiobiology Group, U1296 INSERM, 69008 Lyon, France; nicolas.foray@inserm.fr; 3 Centre Georges François Leclerc, UNICANCER, 27877 Dijon, France; cmirjolet@cgfl.fr; 4 INSERM UMR 1231, Cadir Team, Faculté de Médecine, 27877 Dijon, France; 5 CRAN CNRS UMR 7039, Université de Lorraine, 54505 Vandœuvre-lès-Nancy, France; thierry.bastogne@univ-lorraine.fr; 6 INRIA BIGS, Université de Lorraine, 54505 Vandœuvre-lès-Nancy, France; 7 CYBERnano, Université de Lorraine, 54505 Vandœuvre-lès-Nancy, France; 8 Neolys Diagnostics, 67960 Strasbourg, France; mduclos@neolys-diagnostics.fr; 9 Radiation Therapy Department, Centre Hospitalier Régional Metz-Thionville, 57530 Ars-Laquenexy, France; p.retif@chr-metz-thionville.fr; 10 Centre François Baclesse, 4240 Ezch-sur-Alzette, Luxembourg; guillaume.vogin@Baclesse.lu; 11 UMR 7365 CNRS-UL IMOPA, Équipe 1, Faculté de Médecine, 54505 Vandœuvre-lès-Nancy, France

**Keywords:** cancer, radiation-induced toxicity prediction, biological marker, pATM, normal tissue complication probability

## Abstract

**Simple Summary:**

Early toxicity of radiotherapy (RT) (from the beginning of treatment to 3 months after its end) may compromise cancer treatments and its prediction is a medical, social and economic challenge. Beyond clinical/dosimetric factors, much of the variation in the risk of toxicity is currently unexplained and largely attributed to individual radiosensitivity (IRS). Thus, radiosensitivity tests would help several decision makings for choosing fractionation, preferring surgery to radiation or proton to photons for instance. Numerous initiatives failed in implementing predictive assays of IRS in clinical routine. Here we assess and validate the predictive ability of a new assay RADIODTECT^®^ (RDT), based on phosphorylated ATM protein quantification in lymphocytes, for early toxicity after radiotherapy (RT). This study demonstrated promising results and implementing RTD as an easy managing test in common RT clinical practice for patients can be advisable.

**Abstract:**

Radiation therapy (RT), either alone or in combination with surgery and/or chemotherapy is a keystone of cancers treatment. Early toxicity is common, sometimes leading to discontinuation of treatment. Recent studies stressed the role of the phosphorylated ATM (pATM) protein in RT-toxicity genesis and its ability in predicting individual radiosensitivity (IRS) in fibroblasts. Here we assessed the reliability of the pATM quantification in lymphocytes to predict IRS. A first retrospective study was performed on 150 blood lymphocytes of patients with several cancer types. Patients were divided into 2 groups, according to the grade of experienced toxicity. The global quantity of pATM molecules was assessed by ELISA on lymphocytes to determine the best threshold value. Then, the binary assay was assessed on a validation cohort of 36 patients with head and neck cancers. The quantity of pATM molecules in each sample of the training cohort was found in agreement with the observed Common Terminology Criteria for Adverse Events (CTCAE) grades with an AUC = 0.71 alone and of 0.77 combined to chemotherapy information. In the validation cohort, the same test was conducted with the following performances: sensitivity = 0.84, specificity = 0.54, AUC = 0.70 and 0.72 combined to chemotherapy. This study provides the basis of an easy to perform assay for clinical use.

## 1. Introduction

Radiation-induced toxicity incidence and severity are multifactorial, resulting from a complex interplay of patient- tumor- and treatment-related factors [1,2]. Early toxicity occurs within the first three months after the initiation of the RT and its prediction is a medical, social and economic challenge [3,4]. The use of concurrent chemotherapy improves tumor response to radiation by a variety of mechanisms, including the enhancement of cells response to radiation and especially DNA damage and repairs [5]. Thus, it also increases acute and late toxicities. [6]. Radiosensitivity has been recently defined as any enhanced tissue or cell reaction following exposure to radiation compared to that of normal responding individuals [7]. Beyond clinical/dosimetric factors, most of the variation in the risk of toxicity is currently unexplained and largely attributed to individual radiosensitivity (IRS) [8]. Numerous initiatives failed in implementing predictive assays of IRS in clinical practice and attempted to describe this trait through genetic variants or other assays [9,10].

The biological response to radiation has been proved to depend on recognition and repair of radiation-induced double-strand breaks (RIDSB). Studying the number of micronuclei and the kinetics of both γH2AX and pATM foci post irradiation allows to understand the different steps of the cellular response [11]. From these radiobiological endpoints and since the ATM protein has a major role in RIDSB recognition and repair process [12], a unified model based on radiation-induced ATM nucleoshuttling (RIANS) has been proposed [13]. Any delay in the RIANS process can lead to radiosensitivity and a quantitative correlation was found between the maximal number of nuclear pATM foci assessed by immunofluorescence and the RT-induced toxicities assessed by the Common Terminology Criteria for Adverse Events (CTCAE) severity grades [14]. From this model, two tests that were developed first on skin samples have been shown to reliably predict both IRS and severity of RT adverse events [14,15,16].

Both tests are reliable, however they require a skin sample that patients might be reluctant to provide, and are quite time-consuming.

In order to obtain a faster, clinically pragmatic and less invasive IRS assay, we hypothesized that the overall amount of pATM in patient’s lymphocytes could be representative of their intrinsic ability to repair radiotherapy-related damage and thus of their intrinsic radiosensitivity. We assessed here the reliability of a new approach. This assay, named RADIODTECT^®^, aims at predicting IRS according to the quantification of total pATM on blood lymphocytes by ELISA.

## 2. Materials and Methods

### 2.1. Clinical Data

#### 2.1.1. Training Cohort

A retrospective study was performed on the lymphocytes of 150 patients treated with RT between January 2017 and December 2018 with or without concomitant chemotherapy (Table 1). Patients coming for a follow up consultation during this period, at the earliest 3 months after the end of the radiotherapy, at the latest 6 months after the end of radiotherapy, were proposed to enter the study. The study was approved by the local Ethical Committee. All patients consented to be included in the Neolys Diagnostics collection (2017-A00086-47) and provided a blood sample. Details about patient’s characteristics and treatments are provided in Table 1.

#### 2.1.2. Validation Cohort

Case-series of 36 non-metastatic HNSCC patients treated with postoperative radiotherapy between the 1 January 2017 and the 1 June 2017 with either intensity modulated radiotherapy (IMRT), volumetric modulated arc therapy (VMAT) or tomotherapy, with or without concomitant chemotherapy was considered (Table 2). CTCAE grades data of observed toxicities were prospectively collected for 36 patients of the cohort. Concurrent chemotherapy was cisplatin 100 mg/kg for 7 patients and cisplatin 40mg/kg for 11 patients and 3 patients with TPF (T = docetaxel 75 mg/m², P = cisplatin 75 mg/m², F = 5-fluorouracile 750 mg/m²).

The study was approved by the local Ethical Committee. All patients gave their consent to be included in the Neolys Diagnostics collection (2017-A00086-47) and provided a blood sample during a follow-up consultation.

### 2.2. Toxicity Endpoint Definition

Patients were evaluated for toxicity at baseline, weekly during RT treatment and at RT completion—according to the Common Terminology Criteria for Adverse Events (CTCAE) scale version 4.03. Patients were divided in 2 groups:

- “radioresistant” (RR) patients with early side effects graded <2.
- “radiosensitive” (RS) patients with early side effects graded ≥2.

In the validation cohort, the following three endpoints were specifically considered: mucositis, dysphagia and dermatitis. Indeed, these are the toxicities that are both most frequently observed in patients with HNSCC, and most likely to lead to discontinuation of treatment.

### 2.3. RADIODTECT^®^ Assay

Derived from the RIANS assay [14], this test comprised three steps:

1. Isolation and Treatment of Human Lymphocytes

Lymphocytes were obtained from the blood of patients collected during post-RT follow-up visits and stored at room temperature. Following dilution with equal volume phosphate buffer saline (PBS 1X), blood was poured on 3 mL Ficoll-Paque and centrifuged at 2000× *g* for 20 min. Transferred cells were diluted and then washed twice with PBS, and approximately 1–1.5 × 106 cells were suspended in 1 mL of RPMI1640 containing 10% Fetal Bovine serum (FBS) and 1% antibiotic (penicillin/streptomycin) for use in further tests.

2. Cell Lysis

Cells were pelleted by centrifugation at 500× *g* for 5 min at 4 °C and washed twice with ice-cold PBS1X. The dry pellet was resuspended in 250 µL of RIPA extraction buffer and then incubated on ice for 10 min. The lysed cells were then centrifugated at 15,000× *g* for 15 min at 4 °C, and the supernatant collected in prechilled tubes.

3. ELISA Assay

pATM was quantified in total cell protein fraction by applying an ELISA commercial kit and protocol (#NR-E10877-4, NOVATEIN Biosciences, Woburn, MA, USA) with a similar phosphospecific ser1981pATM antibody which was used for immunofluorescence experiments [12]. The ELISA plates were analyzed with a spectrophotometer (TECAN, Lyon, France) at 450 nm.

### 2.4. Statistical Analysis

#### 2.4.1. Determination of Threshold

The training cohort with 150 patients presenting with different cancer types was used to determine the threshold. After determination of the pATM concentration in the lymphocytes, 150 patients were classified as radiosensitive or radioresistant using the determined cutoff, i.e., 57.8 ng/mL for grade ≥ 2 toxicity.

The receiver operating characteristic (ROC) classification method was used to estimate the optimal pATM threshold and to predict the radiosensitivity classes in the whole cohort. A bootstrap analysis was performed to estimate the average and standard deviation of the classification cut-off. In this bootstrap analysis, the training dataset was composed of 20 RR and 20 RS patients randomly drawn from the initial database composed of 150 cancer patients. We applied the ROC analysis on it and repeated this computation 10,000 times to finally obtain a distribution of the classification cut-off. A Wilcoxon rank sum test was also applied to compare RR and RS groups. ROC analysis and bootstrap analysis were implemented in the R environment for statistical computing (version 3.6.0, 2019-04-26). R Packages used to implement the ROC analysis are pROC (v 1.17.0.1) and caret (v 6.0-86). Performance measures included evaluation of ROC curve and AUC, Chi-Squared test and evaluation of the Odds Ratio for the test in a logistic regression. Statistical analysis was developed on MedCalc statistical software (v. 19.2.6).

The threshold established on the training cohort was then used on the validation cohort.

#### 2.4.2. Performances of the Assay

The discrimination power of the pATM assay with the selected cutoff was evaluated on the validation HNSCC cohort through the area under the ROC curve (AUC). Other performance measures were derived from the confusion matrix. Particularly, (1) Accuracy = (TP + TN)/total (where TP = true positives and TN = true negatives), (2) misclassification rate (or error rate) = (FP + FN)/total (where FP = false positives and FN = false negatives), (3) true positive rate (or sensitivity) = TP/actual positives, (4) false positive rate = FP/actual negatives, (5) true negative rate (or specificity) = TN/actual negatives, (6) precision = TP/predicted positives and (7) null error rate = actual positives/total (i.e., how often one may be wrong if one always predicts the majority class; here, these corresponds to patients showing no toxicity).

The results of the pATM quantification were then combined with the chemotherapy use, which we simplified into a binary variable (concomitant chemotherapy = 1; no concomitant chemotherapy = 0) and a logistic regression modeling was performed, with an evaluation of the AUC.

## 3. Results

### 3.1. Population

One hundred and fifty patients were available for analysis on the training cohort and thirty-six HNSCC patients for the validation cohort. Patients’ characteristics and oncologic treatment for the training and validation cohort are reported in Table 1 and Table 2 respectively.

### 3.2. pATM Threshold Determination

Grade ≥ 2 early toxicity was experienced by 61/150 (40%) patients in the training cohort. Among them, 22 had received concurrent chemotherapy.

**Figure 1:**
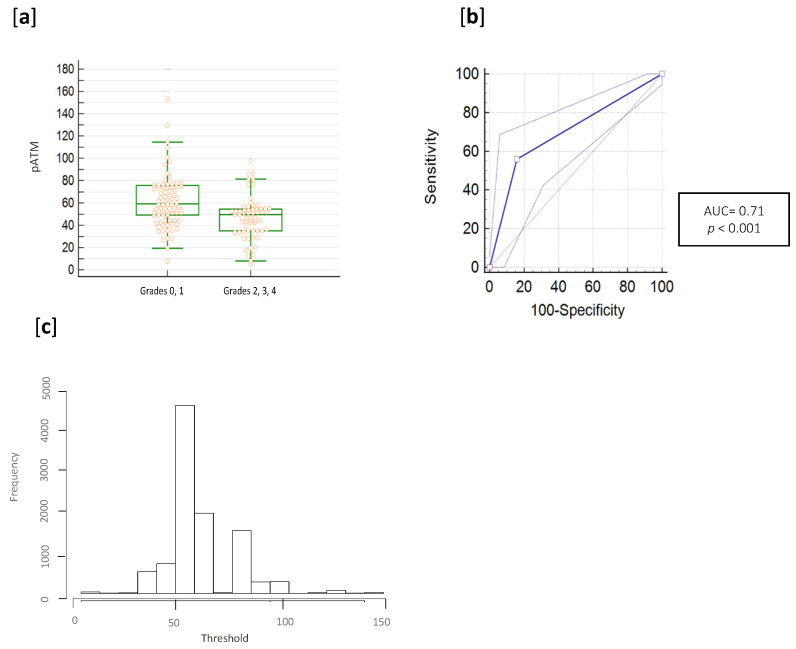
Determination of the optimized threshold and prediction results of the 150 patients. (**a**) pATM ELISA assay distribution (pATM quantities are in ng/mL). (**b**) ROC analysis was performed on pATM ELISA results and a Wilcoxon rank sum test was also applied to compare RR and RS groups (*p* < 0.001). (**c**) Histogram of threshold frequencies after the bootstrap analysis.

The distribution of pATM concentrations observed on the training cohort is shown in Figure 1a for patients with and without toxicity (*p*-value for the Chi-square test *p* < 0.0001), while Figure 1b reports the ROC curve with an AUC of 0.71 (95% Confidence Interval: 0.63–0.77) with negative predictive value (NPV) of 0.53 and positive predictive value (PPV) of 0.85.

The optimal threshold was estimated at 57.8 ng/mL (with bootstrapping) with an empirical interquartile range equal of 54.79–64.39 ng/mL (Figure 1c for distribution of pATM concentration cutoffs in the 10,000 bootstrap resamples). Odds ratio (OR) obtained for the assay was 6.7 with an interquartile range of 2.9 to 15.3.

Using the best estimated cutoff, 48 and 52 patients were well-classified as RR and RS. Results for the metrics calculated from the confusion matrix were: accuracy = 0.67, misclassification rate (or error rate) = 0.33, true positive rate (or sensitivity) = 0.85, true negative rate (or specificity) = 0.54, precision = 0.85, null error rate = 0.40.

### 3.3. RADIODTECT^®^ Assay Performances on Validation Cohort

Grade ≥ 2 early toxicity was experienced by 25/36 (69%) patients in the HNSCC cohort. Among them, 20 received concurrent chemotherapy.

**Figure 2:**
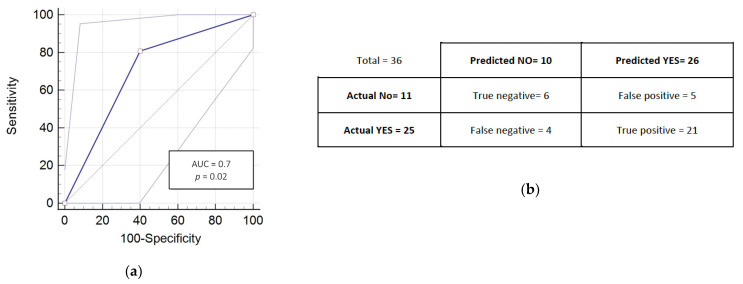
Prediction results and performances of the RadioDtect^®^ assay of the 36 HNSCC patients. (**a**) ROC analysis was performed on pATM ELISA results and a Wilcoxon rank sum test was also applied to compare RR and RS groups (*p* = 0.02). (**b**) Confusion matrix results for the prediction from the pATM data.

Figure 2 reports the ROC curve with an AUC = 0.7 (95% confidence interval: 0.53-0.84) (Figure 2a) and the confusion matrix for prediction of grade ≥2 early toxicity (Figure 2b); Using the same cutoff, 11 and 25 patients were well-classified as RR and RS. Results for the metrics calculated from this matrix were: accuracy = 0.75, misclassification rate (or error rate) = 0.25, true positive rate (or sensitivity) = 0.84, true negative rate (or specificity) = 0.54, precision = 0.80, null error rate = 0.69.

### 3.4. Prediction of Radiosensitivity Considering RadioDtect and the Addition of Concurrent Chemotherapy as a Binary Variable

Chemotherapy alone was not significantly associated to Grade ≥ 2 in the two cohorts (Fisher’s exact test *p* = 0.5 and *p* = 0.15 for the testing and validation cohorts respectively).

**Figure 3:**
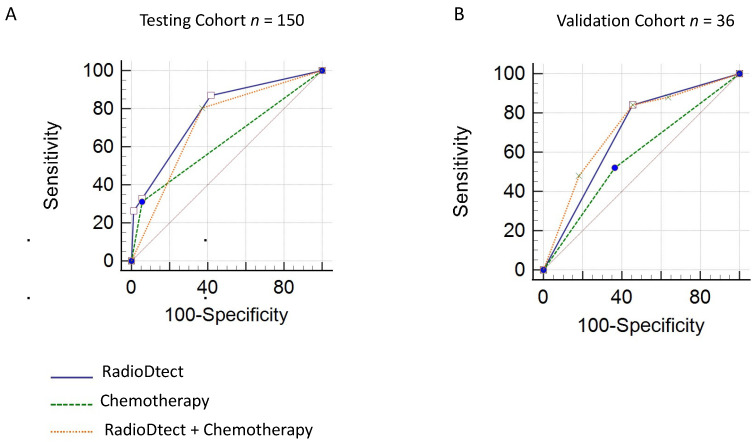
Performance analysis of combination of RadioDtect and chemotherapy as a binary variable for predicting grade ≥ 2 toxicities (**A**) ROC analysis for the testing cohort (**B**) ROC analysis for the validation cohort.

Associating concurrent chemotherapy as a variable to RadioDtect data improves the assessment of radiosensitivity in the two cohorts with a gain of 0.06 in AUC for the testing cohort (AUC = 0.77, *p* ≤ 0.001) and a gain of 0.2 for the validation cohort (AUC = 0.72, *p* = 0.06) (Table 3 and Figure 3).

## 4. Discussion

Predicting radiosensitivity is a key step in the personalization of radiotherapy treatments [6].

It has been showed that RIANS-based model allows to predict patients radiosensitivity in different situations [13]. Firstly, it is noteworthy that the RIANS model has provided relevant biological interpretation of the radiosensitivity associated with genetic syndromes [17,18,19]. Secondly, the RIANS model was shown to provide a relevant biological interpretation to the relative biological efficiency/linear energy transfer relationship by using a large spectrum of radiation and particle types including protons [19]. Finally, the RIANS model was also shown to provide biological interpretation of the alpha and beta parameters of the linear-quadratic model on which NTCP approach is generally based [12]. Altogether, these data suggest that RIANS model is a strong mechanistic model to explain the individual response to radiation in many situations [13]. From a biological point of view, a quantitative correlation was found between the maximal number of nuclear pATM foci assessed by immunofluorescence after irradiation and the severity of the RT-induced toxicities according to CTCAE severity grades [14].

However, due to the cellular amplification step, the RIANS assay requires 1–3 weeks to get a result. This delay makes it unsuitable for using it in clinical practice, as decisions must be taken early to avoid delaying treatments. To shorten the delays, a predictive assay quantifying the pATM proteins via an enzyme-linked immunosorbent assay (ELISA) has already been reported on skin fibroblasts [16]. In this study, pATM proteins can be detected and quantified at basal level in fibroblasts of radiosensitive patients [RS] and predict radiosensitivity without requiring irradiation [16]. Indeed, the mandatory irradiation of the sample for RIANS assay is a time-consuming step. To reduce the time between the patient’s sample collection and the result, and use a less invasive sample, we assessed here a predictive model based on the quantification of pATM in blood lymphocytes. Indeed, lymphocytes are nucleated cells in which pATM is expressed [20], and can be easily extracted from a blood sample. The model derived from the RIANS was based on preliminary results suggesting that overall amount of pATM did not change in lymphocytes after irradiation in patients with genetic syndromes (WO2018229439A1) that’s why we evaluated lymphocyte pATM quantity without irradiation in this new assay.

In our study we demonstrated and validated in two different cohorts the ability of the pATM ELISA quantification to predict radiation toxicities. pATM assay predicts radio-induced toxicities independently to sex patient while no significant difference of pATM has been highlighted between male and female using training cohort (Pereira, S. Inserm URM 1296, France, Unpublished work, 2020).

We observed that the AUC of the RADIODTECT^®^ assay is a little lower than the immunofluorescence RIANS based assay (71% vs. 85%).

Its discriminative power was improved when taking into account the use of concurrent chemotherapy as a binary data. Gain in AUC for the combined models ranged from 2% to 5% (for the validation and the testing cohorts respectively). This suggests that taking into account both the use of chemotherapy as a radiosensitizer and the individual intrinsic capacity to repair DNA damage can be responsible to the final response to an individual treatment. The use of concurrent chemotherapy should therefore be taken into account to evaluate the risk of toxicity, using it as an adjustment variable as its weight is minor compared to the RIANS variable. However, in our study, the main limitation is the small proportion of patients who received concurrent chemotherapy: the results of this pilot study should thus be considered as a proof of concept and should be consolidated with other experiments, and larger cohorts.

It is noteworthy that all approaches based on or derived from the RIANS model allow to predict radiation toxicities independently of the type of cancer and of reactions [14,16].

Different types of tests predicting the occurrence of radio induced toxicities in normal tissue have been described using lymphocytes, based on the study of their apoptosis or genomic signatures [21,22,23,24]. In these assays as well, the predicted radiosensitivity classifies patients into two groups corresponding to severity grade ≥ 2 and grade < 2. Some studies require the irradiation of the sample [21,22,25]. Most of them are designed to predict late side effects [21,25,26,27], and only a minority are focused on the prediction of acute side effects [22]. Moreover, despite similarities in the experimental protocols, the observed AUC is higher for the pATM ELISA assay compared to others [16,21,22,23].

## 5. Conclusions

This study is a proof of concept, demonstrating promising results as this pATM quantification-based assay can predict the risk of early toxicity with an AUC = 71%, regardless of the location of the irradiation. This new assay is intended for routine use in clinical practice one day. However, cutoff values established in the present analysis has to be validated in a larger prospective cohort. Furthermore, RadioDtect results could be further refined by combining them with models taking into account the dosimetric parameters, and by specifying the effects of the radiosensitizers to which the patient is exposed.

## 6. Patents

N.F. and S.P. reports the following patents: FR3040178A1, FR3040179A1, WO2017098190A1, EP3685163A1.

## Figures and Tables

**Table 1:** Training cohort: 150 patients’ characteristics and treatments.

Patients’ Characteristics and Treatments		Head and Neck Patients *N* = 53	Prostate Patients *N* = 63	Breast Patients *N* = 24	Rectum Patients *N* = 5	Others (Brain, Lung, Lymph Node, Esophagus) *N* = 5	Total *N* = 150
Gender	Female	13	0	24	3	3	43
	Male	40	63	0	2	2	107
Median Age (Range)		61 (31, 84)	64 (32, 83)	69 (49, 89)	59 (49, 69)	66.5 (56, 77)	62.5 (31; 84)
Type of Radiotherapy	Definitive	4	52	0	2	1	59
	Adjuvant	49	11	24	3	4	91
Mean Dose	Tumor (range)	62 (50–70)	70 (62–80)	54.2 (42.4–66)	47.4 (36–59.4)	45 (24–66)	52 (24–80)
Concurrent Chemotherapy	Yes	19 (Cisplatin)	0	0	3 (2 capecitabin mitomycin, 1 Cisplatin)	2 (1 Folfox, 1 carboplatin, 5FU)	24
	No	34	63	24	2	3	126
Concurrent Hormonotherapy	Yes	-	16	-	-	-	16
	No		47				134
CTCAE Highest Score	1	17	55	14	1	1	89
	2	20	7	8	2	1	38
	3	13	1	2	2	2	20
	4	3	0	0	0	0	3

CTCAE: Common Terminology Criteria for Adverse Events.

**Table 2:** Validation cohort: 36 patients’ characteristics and treatments.

Patients’ Characteristics and Treatments		Head and Neck Patients *N* = 36
Gender	Female	4
	Male	32
Median Age (Range)	-	57 (32–85)
Type of radiotherapy	VMAT	28
	IMRT	7
	Tomotherapy	1
Mean Dose	Tumor (range)	60 Gy (50–70 Gy)
Concurrent Chemotherapy (Cisplatin and TPF)	Yes	20
	No	16
CTCAE Highest Score for Acute Toxicities	1	11
	2	11
	3	10
	4	4

**Table 3:** Result on performance of RadioDtect and combined RadioDtect + chemotherapy approach for Grade ≥ 2 toxicities.

Performances	Training Cohort		Validation Cohort	
	RadioDtect	RadioDtect + Chemotherapy	RadioDtect	RadioDtect + Chemotherapy
AUC	0.71	0.77	0.70	0.72
95% CI	0.63 to 0.77	0.70 to 0.84	0.53 to 0.84	0.59 to 0.85
*p*-value	<0.001	<0.001	0.02	0.06

## Data Availability

The datasets generated for this study are available on request to the corresponding author.
